# Macrophage migration inhibitory factor -173 G/C polymorphism is associated with an increased risk of pulmonary tuberculosis in Zahedan, Southeast Iran

**DOI:** 10.17179/excli2014-636

**Published:** 2015-01-21

**Authors:** Mohammad Hashemi, Batool Sharifi-Mood, Azam Rasouli, Shadi Amininia, Mohammad Naderi, Mohsen Taheri

**Affiliations:** 1 Cellular and Molecular Research Center, Zahedan University of Medical Sciences, Zahedan, Iran; 2 Department of Clinical Biochemistry, School of Medicine, Zahedan University of Medical Sciences, Zahedan, Iran; 3 Research Center for Infectious Diseases and Tropical Medicine, Zahedan University of Medical Sciences, Zahedan, Iran; 4 Genetics of Non Communicable Disease Research Center, Zahedan University of Medical Sciences, Zahedan, Iran

**Keywords:** tuberculosis, macrophage migration inhibitory factor, MIF, polymorphism

## Abstract

Macrophage migration inhibitory factor (MIF) has an important role in controlling infection. The aim of this study was to evaluate the possible association between *MIF* -173 G/C functional polymorphism and pulmonary tuberculosis (PTB) in an Iranian population from Zahedan Southeast Iran. This case-control study was done on 161 PTB and 142 healthy subjects. Genomic DNA was extracted from all participants by salting out method. The *MIF* -173 G/C variant was genotyped using polymerase chain reaction-restriction fragment length polymorphism (PCR-RFLP). The finding showed that the *MIF* -173 G/C polymorphism increased the risk of PTB in codominant (OR=1.76, 95 % CI=1.05-2.95, p=0.038, GC vs GG) and dominant (OR=1.78, 95 % CI=1.09-2.91, p=0.027, GC+CC vs GG) tested inheritance models. Furthermore, the minor allele frequency (MAF) increased the risk of PTB in comparison with G allele (OR=1.63, 95 % CI=1.07-2.48, p=0.028). In conclusion, the present study provides evidence that -173 G/C polymorphism may increase the risk of PTB.

## Introduction

Tuberculosis (TB) is caused by the *bacillus Mycobacterium tuberculosis *and remains a major global health problem especially in Asia and Africa (Orcau et al., 2011[27]). According to the report of World Health Organization, approximately 8.6 million new cases of TB were reported in 2012 (Zumla et al., 2013[36]). Though one-third of population is infected with TB, merely 10 % of infected cases will develop clinical disease during their lifetime. Multiple factors contribute to the risk of infection and development of TB including environmental factors, host-pathogen interactions and genetic factors (Bellamy, 2003[7]). Increasing evidence indicates that the risk of developing tuberculosis in human is strongly influenced by genetic factors (Azad et al., 2012[3]).

Human macrophage migration inhibitory factor (MIF*)* gene is located on chromosome 22q11.2. A functional variant in within the 5` promoter region of *MIF*, located at position -173 replacing G to C, appears to affect promoter activity in a cell-type dependent manner (Donn et al., 2002[16]; Renner et al., 2005[29]). *MIF* encodes a multifunctional cytokine, MIF, which is produced by several types of cells, including epithelial cells and cells that participate in the innate and adaptive immune responses (Bacher et al., 1997[4]; Calandra et al., 1994[10]). MIF protein is a pleiotropic cytokine produced by activated T cells, macrophages, and the pituitary gland (Bernhagen et al., 1993[8]; Bloom and Bennett, 1966[9]; Calandra et al., 1994[10]). 

MIF is considered an immuno-regulatory cytokine, and the role of MIF during microbial infection has been recognized to its ability to initiate an innate immune response by inducing tumor necrosis factor-α (TNF-α) production and other pro-inflammatory cytokines (Marinho et al., 2007[23]; Roggero et al., 2002[30], 2004[31]). 

It has been proposed that gene variants play an important role in the occurrence and development of TB (Bahari et al., 2012[5]; Naderi et al., 2013[24], 2014[25]). Several studies have shown that *MIF* -173 G/C variant increased the risk of TB (Gomez et al., 2007[17]; Li et al., 2012[21]; Sadki et al., 2010[32]). To the best of our knowledge, there is not any report regarding the impact of this variant on TB in Iranian population. Therefore, the present study was designed to find out the possible association between *MIF* -173 G/C variant and PTB in a sample of Iranian population.

## Materials and Methods

This case-control study was done on 161 PTB patients and 142 population-based healthy subjects. The subjects who underwent PTB treatment and newly diagnosed PTB cases were enrolled in the study within the case group. The diagnosis of PTB was based on clinical, radiological, sputum Acid Fast Bacillus (AFB) smear positivity, culture, and response to antituberculosis therapy as described previously (Hashemi et al., 2013[18]; Naderi et al., 2014[26]). Control subjects were selected from the Zahedan population showing no recent signs, symptoms, or history of pulmonary infections. The local Ethics Committee of the Zahedan University of Medical Sciences approved the project, and written informed consent was taken from all individuals. Genomic DNA was extracted from whole blood using salting out method as described previously (Hashemi et al., 2010[19]). 

Genotyping of rs755622 polymorphism of *MIF* was done by PCR-RFLP methods. The forward and reverse primers were 5`- CTCAAACACACAAGCTCACGCATGCG-3` and 5`-ACCACTGTGGTCCCGCCTTTTG TGAC-3`, respectively. In each 0.20 ml reaction, 1 µl of genomic DNA (~100 ng/ml), 1 µl of each primers and 10 µl of 2X Prime Taq Premix (Genet Bio, Korea) and 7 µl ddH_2_O were added. The PCR conditions were set as follows: 95 °C for 5 min, 30 cycles of 95 °C for 30 s, 65 ° for 30 s, and 72 °C for 30 s and a final extension step of 72 °C for 10 min. Ten microliter of PCR product digested by AluI restriction enzyme. The C allele digested and produces 255bp and 184bp while the G allele undigested and produce 439bp fragment. 

### Statistical analysis

Statistical analysis was done using statistical package SPSS 18 software (SPSS for Windows (SPSS, Inc., IL, USA). Data were analyzed by independent sample t-test and χ2 test. The associations between MIF polymorphism and PTB were assessed by computing the odds ratio (OR) and 95 % confidence intervals (95 % CI) from logistic regression analyses adjusted for sex and age. A p-value < 0.05 was considered statistically significant. We estimated the Hardy-Weinberg equilibrium (HWE) separately for cases and controls.

## Results

The study group consists of 161 PTB patients (61 male, 100 female) and 142 healthy subjects (62 male, 80 female). The mean age of PTB, and healthy individuals were respectively 50.6 ± 20.5, and 47.3 ± 15.4 years. No significant difference was found between the groups regarding sex and age (p > 0.05). 

The finding showed that the *MIF* -173 G/C polymorphism increased the risk of PTB in codominant (OR=1.76, 95 % CI=1.05-2.95, p=0.038, GC vs GG) and dominant (OR=1.78, 95 % CI=1.09-2.91, p=0.027, GC+CC vs GG) tested inheritance models. 

Furthermore, the minor allele frequency (MAF) of MIF -173 G/C increased the risk of PTB in comparison with G allele (OR=1.63, 95 % CI=1.07-2.48, p=0.028). 

The genotype of MIF -173 G/C variant in controls and cases were in HWE (χ2=1.59, P=0.207 and χ2=0.289, P=0.590, respectively) (Table 1(Tab. 1)).

## Discussion

It has been proposed that the MIF is a critical mediator of the innate immune response to TB (Das et al., 2013[14]). In the present study we investigated the impact of *MIF* -173 G/C (rs755622) functional variant on PTB risk in a sample of Iranian population. Our findings suggest that rs755622 GC as well as GC+CC genotypes increased the risk of PTB. Also, subjects with *MIF* rs755622 C allele were found to be more susceptible to PTB.

Gomez et al. (2007[17]) reported that *MIF* -173 C allele increased the risk of TB in Colombian population. Li et al. (2012[22]) investigated the association between human *MIF* promoter polymorphism and tuberculosis in a Southwestern Chinese population. They found that the *MIF* -173 G/C polymorphism increased the risk of TB (OR=2.12, 95 % CI=1.45-3.10 GC+CC vs. GG). Sadki et al. (2010[32]) have found a statistically significant increase of the *MIF* -173 CC homozygote genotype and MIF -173 C allele frequencies in PTB patients compared with healthy controls in Moroccan population. It has been shown that *MIF* -173 G/C polymorphism is not associated with incidence of pulmonary hamartoma (Kaznowska et al., 2007[20]). 

It has been shown that serum level of MIF was significantly higher in patients with pulmonary tuberculosis than in controls (Li et al., 2012[22]; Yamada et al., 2002[35]). High levels of serum MIF and interleukin 10 (IL-10) are shown to be associated with a rapidly fatal outcome in patients with severe sepsis (Chuang et al., 2014[13]).

Mycobacterium tuberculosis mainly infects macrophages *in vivo*, which can result in substantial macrophage activation, proliferation, recruitment to the site of infection, aggregation, invasion, and secretion of several cytokines to produce a strong immune response which may also cause tissue damage. MIF plays an essential role in the pro- and anti-inflammatory response to infection. 

Earlier studies have shown that polymorphisms in promoter influence the basal and/or induced transcription activity of MIF, and the -173 C allele is associated with greater production of MIF protein (Donn et al., 2002[16]). Higher reporter luciferase activity for both the *MIF* -173 C allele and the *MIF* CATT7-173C haplotype has been founded in a human T-lymphoblast cell line (Baugh et al., 2002[6]).

MIF is considered an integral component of the host antimicrobial alarm system and stress response that promotes the pro-inflammatory functions of immune cells (Calandra et al., 2003[11]). MIF serum levels are significantly increased in parasitic diseases such as leishmaniasis and malaria (Awandare et al., 2007[1]; Chaiyaroj et al., 2004[12]), and functional polymorphism of the human *MIF* gene has been associated with increased susceptibility or severity to inflammatory (de Jong et al., 2001[15]; Renner et al., 2005[29]), autoimmune (Radstake et al., 2005[28]; Sanchez et al., 2006[33]; Stosic-Grujicic et al., 2009[34]), and infectious diseases (Awandare et al., 2006[2]; Gomez et al., 2007[17]).

In conclusion, our finding provides evidence that *MIF* -173 G/C functional polymorphism may contribute to the risk of PTB in a sample of Iranian population. Validation by a larger prospective study from an additional diverse ethnic population is necessary to verify the results.

## Acknowledgements

This work was supported by dissertation grant from Zahedan University of Medical Sciences.

## Conflict of interest

The authors declare no conflicts of interest.

## Figures and Tables

**Table 1:**
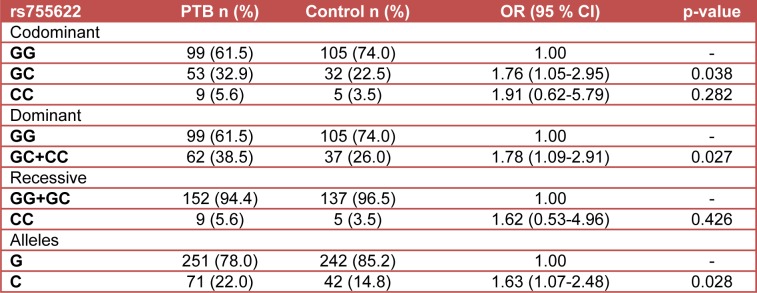
Comparison of genotype frequency of *MIF *-137 G/C (rs755622) polymorphism in PTB and controls
